# Long-Term Survival After Definitive Concurrent Chemoradiation Therapy for Synchronous Small Cell Neuroendocrine Carcinoma of the Urinary Bladder and Adenocarcinoma of the Prostate: A Case Report

**DOI:** 10.7759/cureus.51481

**Published:** 2024-01-01

**Authors:** Michael T Hsieh, Richard Tustin, Tue Le, Abdul Rahim Mohd Tahir, Thomas P Shakespeare

**Affiliations:** 1 Radiation Oncology, Coffs Harbour Health Campus, Mid North Coast Local Health District, New South Wales, AUS; 2 Anatomical Pathology, Coffs Harbour Health Campus, Mid North Coast Local Health District, New South Wales, AUS; 3 Radiation oncology, Coffs Harbour Health Campus, Mid North Coast Local Health District, New South Wales, AUS

**Keywords:** prostate adenocarcinoma (pca), muscle-invasive bladder cancer, prostate cancer, definitive radiotherapy, concurrent chemoradiotherapy, concurrent chemoradiation therapy, synchronous primary cancers, poorly differentiated neuroendocrine carcinoma, small cell carcinomas, small cell bladder carcinoma

## Abstract

Available reports of synchronous prostate and bladder cancer have exclusively described radical cystoprostatectomy with or without perioperative chemotherapy as the treatment of choice. There are no reports of curative intent or definitive chemoradiation therapy for synchronous primary bladder and primary prostate cancers. Small cell carcinoma of the bladder is a rare and aggressive tumor. We present the first case of synchronous mixed small cell carcinoma and urothelial carcinoma of the urinary bladder and adenocarcinoma of the prostate in a 70-year-old male who attained long-term survival after curative intent and definitive concurrent chemoradiotherapy with minimal acute and late toxicities. The patient remained alive and disease-free at 41 months post-treatment and achieved excellent functional outcomes with organ preservation. Definitive chemoradiation therapy offers a safe and effective, curative-intent organ preservation treatment for localized synchronous prostate and bladder cancers.

## Introduction

Small cell carcinoma of the urinary bladder is a rare and aggressive tumor, comprising 0.7%-1% of all bladder cancer diagnoses, and of all the bladder cancer subtypes, it has the worst prognosis [1,2]. Synchronous prostate and bladder cancer is most commonly encountered in radical cystoprostatectomy series for primary bladder cancer [3]. There is no MEDLINE (Medical Literature Analysis and Retrieval System Online)-indexed reports to date of synchronous small cell carcinoma of the bladder or prostate adenocarcinoma treated with definitive chemoradiation therapy. Furthermore, there are no published reports to date of definitive chemoradiation therapy for synchronous urothelial carcinoma and prostate cancer. We herein present the first case of synchronous mixed small cell carcinoma and urothelial carcinoma of the urinary bladder and adenocarcinoma of the prostate, treated with curative intent and definitive concurrent chemoradiation therapy with minimal toxicity, resulting in excellent functional outcomes and organ preservation with long-term survival.

## Case presentation

Clinical presentation

**Figure 1:**
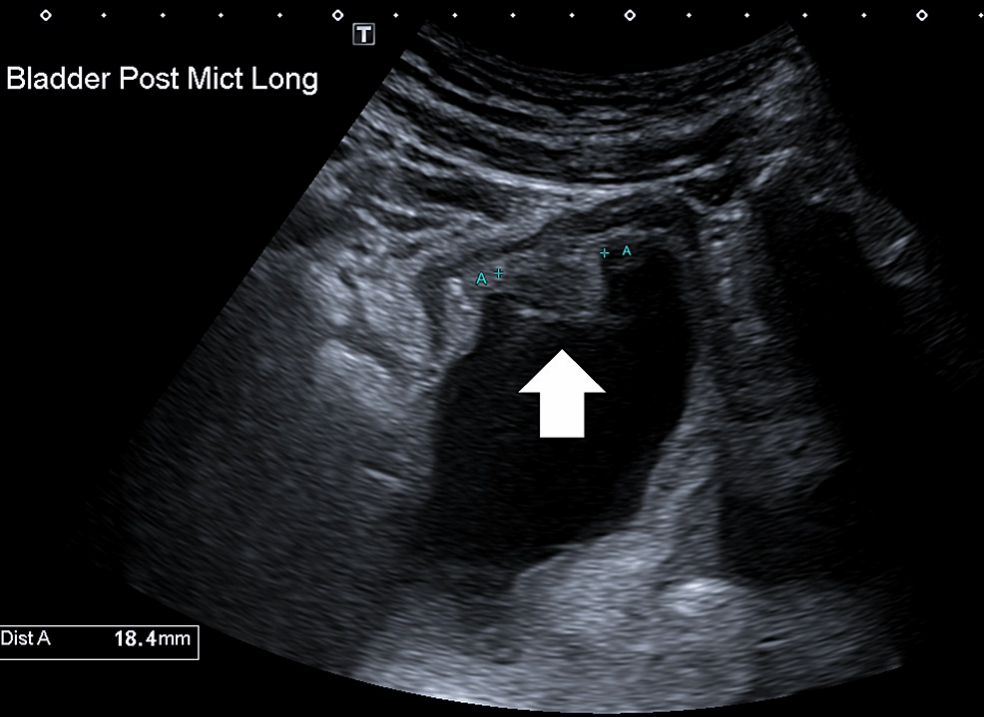
Ultrasound of the bladder: post-micturition, longitudinal view. An 18-mm mass was noted at the anterior bladder wall.

A 70-year-old male, Eastern Cooperative Oncology Group performance status score (ECOG) 1, ex-smoker, medically fit apart from ischemic heart disease and previous coronary artery bypass graft and peripheral vascular disease requiring femoral artery angioplasty, presented to his family physician with severe obstructive lower urinary tract symptoms in November 2019. A digital rectal examination found apical prostate nodularity. Prostate-specific antigen (PSA) was elevated at 5.6 ug/L. He was referred for an ultrasound of the urinary tract, which demonstrated a 14 x 15 x 18mm lesion on the right anterior bladder wall, associated with gross thickening of the bladder wall, and an elevated postvoid residual of 106 cm with no hydroureter or hydronephrosis (Figure 1). Urine cytology showed atypical cells in all three samples submitted. Baseline urinary symptoms included nocturia x 6, Common Terminology Criteria for Adverse Events version 5 (CTCAE v5), grade 2 urinary urge, and grade 1 urinary retention. He denied urinary incontinence or hemorrhagia. Bowel and sexual functions were reportedly normal. There was no family history or personal history of malignancies, and there was no previous radiation therapy. He was referred to a urologist for further workup. 

Investigations

**Figure 2:**
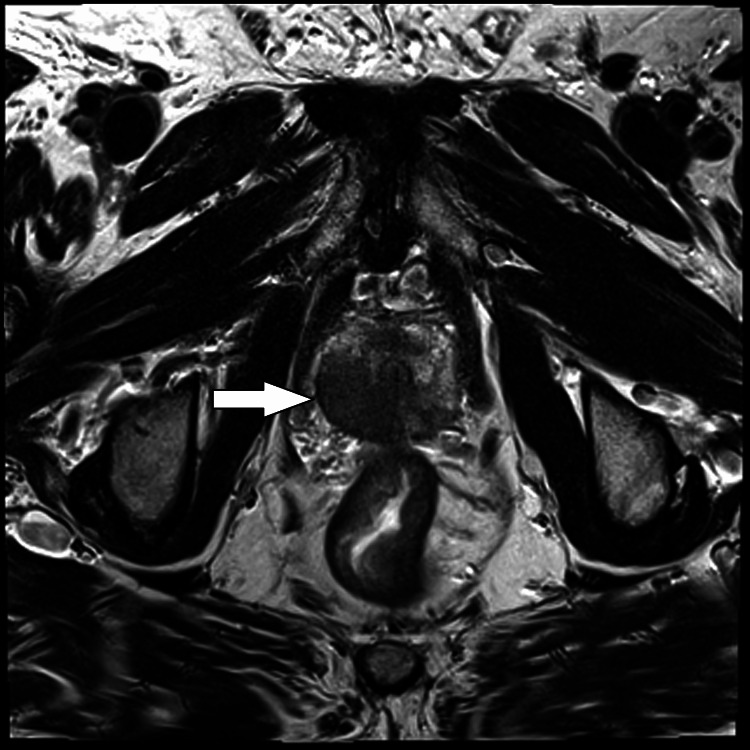
Multiparametric magnetic resonance imaging of the prostate: 30mm PI-RADS 5 lesion with characteristic T2 hypointensity involving the right peripheral zone and transition zone from base to apex.

**Figure 3:**
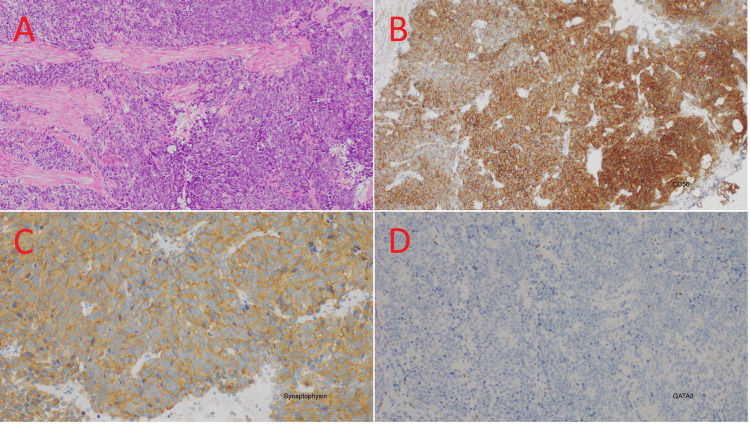
Histopathology and immunoperoxidase stain (IHC) of small cell carcinoma of the bladder in this patient. (A) Hematoxylin and eosin. Sheets to nests of high-grade tumor cells with neuroendocrine differentiation invading muscle. (B) CD56 IHC-positive. (C) Synaptophysin IHC is positive. (D) GATA3 IHC is negative.

**Figure 4:**
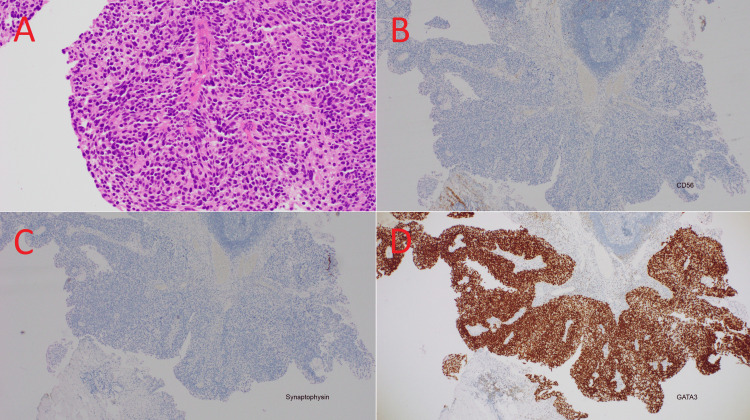
Histopathology and immunoperoxidase stain (IHC) of high-grade papillary urothelial carcinoma of the bladder in this patient. (A) Hematoxylin and eosin. Malignant cells with classic “mosaic tile” urothelial differentiation, organized around a fibrovascular core. (B) CD56 IHC is negative. (C) Synaptophysin IHC is negative. (D) GATA3 IHC positive.

**Figure 5:**
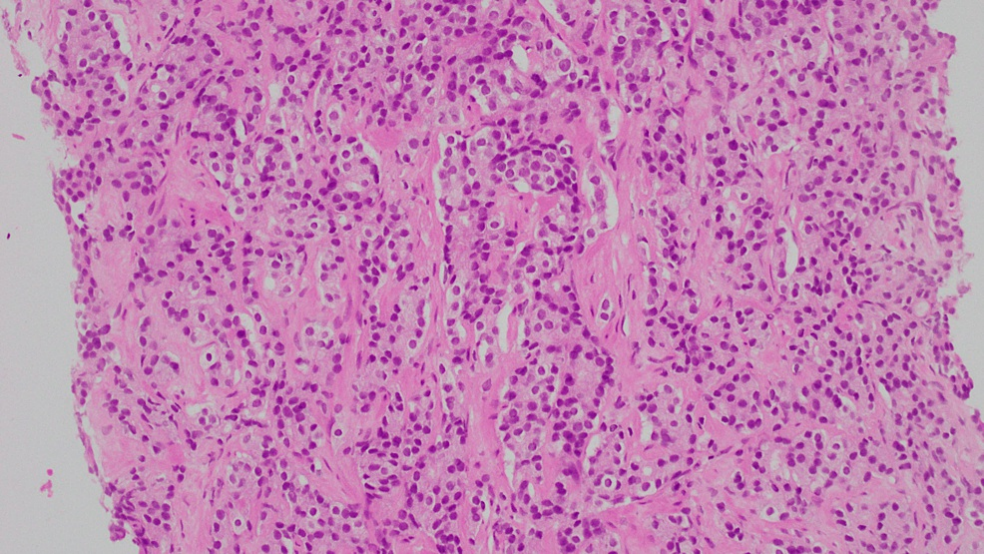
Histopathology of prostate core biopsies, hematoxylin, and eosin: a mix of pattern 5 and 4 prostate acinar adenocarcinomas, with no neuroendocrine differentiation.

Multiparametric magnetic resonance imaging of the prostate found a non-enlarged prostate measuring 23.4cc and a 30mm PI-RADS 5 (prostate imaging reporting and data system) lesion, extending from base to apex across the right peripheral zone and transition zone, with no overt extraprostatic extension or seminal vesicle invasion (Figure 2). A transurethral resection of the bladder tumor was performed in February 2020. Histopathology from the resection specimen showed both muscle-invasive mixed small cell carcinoma and high-grade papillary urothelial carcinoma. The small cell component showed archetypal immunoperoxidase staining for neuroendocrine differentiation, with strong expression of neuroendocrine markers CD56 and synaptophysin and a lack of expression of GATA3, a marker for urothelial differentiation (Figures 3A-3D). The muscle-invasive high grade papillary urothelial carcinoma lacked expression of both neuroendocrine markers and expressed GATA3 strongly (Figures 4A-4D). The small-cell neuroendocrine component comprised >80% of the tumor volume. One month later, a transrectal ultrasound-guided core biopsy of the prostate demonstrated high-grade acinar adenocarcinoma, Gleason 5+4=9 in 11 out of 13 cores, ISUP (International Society of Urological Pathology) Grade Group 5, with perineural invasion and intraductal carcinoma, but no evidence of neuroendocrine differentiation (Figure 5). The reporting pathologist stated that the prostatic adenocarcinoma was a separate primary cancer from the small cell carcinoma of the bladder (SCCB).

**Figure 6:**
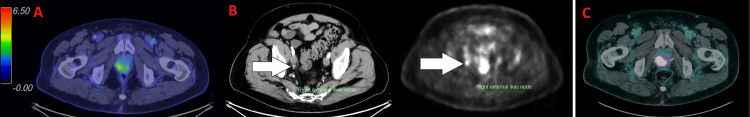
Staging positron emission tomography (PET) scans. (A) Fludeoxyglucose-PET uptake in the prostate primary, SUV max 6. (B) Fludeoxyglucose-PET uptake in a right external iliac lymph node, SUV max 4.2, PET, and co-registered computer tomography scans presented side by side. (C) Gallium-68 prostate-specific membrane antigen-PET uptake in the prostate primary, SUV max 27.

Staging contrast-enhanced computed tomography (CT) of the brain did not reveal any brain metastases. An MRI of the brain was not performed on this patient. Staging fludeoxyglucose positron emission tomography (FDG-PET) showed intense right-sided prostatic uptake from base to apex, SUV max 6.0 (Figure 6A). Prostate-specific membrane antigen positron emission tomography (PSMA-PET) also showed intense right-sided prostatic uptake from base to apex, SUV max 27.0 (Figure 6C). There were no nodal or distant metastases on PSMA-PET. A right external iliac lymph node was moderately FDG-PET avid, SUV max 4.2 (Figure 6B), but demonstrated minimal PSMA-PET avidity (SUV max 1.8). His case was reviewed at the local urology multidisciplinary tumor board, and the right external iliac FDG-PET-avid lymph node was thought to represent metastatic spread from SCCB. Thus, he was formally staged with T2N1M0 bladder cancer and NCCN (National Comprehensive Cancer Network) high-risk prostate cancer, T2N0M0. The tumor board recommended perioperative systemic therapy, radical cystoprostatectomy, and pelvic lymph node dissection. The tumor board recommended against prophylactic cranial irradiation. The patient declined surgery and requested organ preservation with definitive chemoradiation therapy. 

Treatment

**Figure 7:**
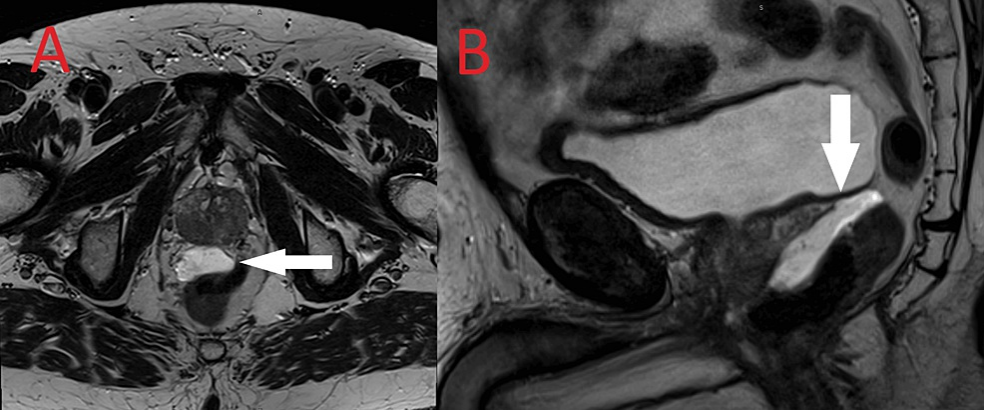
Prostate magnetic resonance imaging is used for radiotherapy planning. A hyperintense T2 signal is seen in the spaceOAR barrier gel inserted between the rectum and prostate. (A) Axial view. (B) Sagittal view.

**Figure 8:**
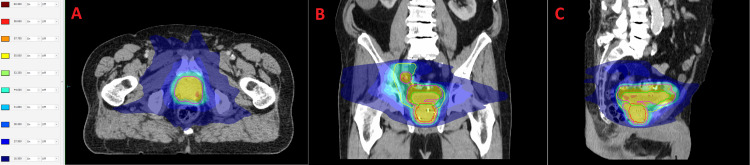
Dose distribution and target volumes of the treatment plan employing Volumetric Modulated Arc Therapy. Pink line: High risk planning target volume. Yellow line: Low risk planning target volume. Green line: Bladder. Red line: Prostate. Isodoses are presented in dose-wash. (A) Axial slice at the level of the prostate. Absolute dose (Gy) of isodose lines is displayed on the left. (B) Coronal slice. (C) Sagittal slice.

He was prescribed curative intent, definitive radiation therapy with concurrent chemotherapy, volumetric modulated arc therapy, and a simultaneous integrated boost to two dose levels: 55 Gy in 20 fractions for the high-risk planning target volume (PTV-HR) and 44 Gy in 20 fractions for the low-risk planning target volume (PTV-LR), delivered in five fractions per week over four weeks. The patient was commenced on neoadjuvant androgen deprivation therapy (ADT) and recommended for two years of adjuvant ADT as tolerated (Leuprorelin 22.5mg every three months). He underwent prostate fiducial seed and barrier gel placement and an MRI prostate for radiotherapy planning (Figures 7A-7B). At CT simulation, he was positioned supine with an empty bladder and rectum. A CT with 2mm slices was acquired of the pelvis. Diagnostic FDG-PET, PSMA-PET, and a dedicated planning MRI of the pelvis were fused to aid contouring. An involved-field radiation strategy was employed, treating bladder, prostate, and ipsilateral pelvis only, in keeping with the treatment paradigm for limited-stage small-cell lung cancer. The high-risk clinical target volume (CTV-HR) included the bladder, prostate, and the FDG-PET-avid right external iliac lymph node. The low-risk clinical target volume (CTV-LR) included seminal vesicles and elective right internal iliac and external iliac nodal stations. A uniform 7mm expansion from CTV-HR and CTV-LR was used to create the respective planning target volumes, PTV-HR and PTV-LR. Dose distribution and target volume contours are represented in Figures 8A-8C. The medical oncologist prescribed four cycles of three-weekly concurrent cisplatin and etoposide (intravenous cisplatin 60 mg/m2 day one, intravenous etoposide 120 mg/m2 days 1 to 3). 

Outcome

The patient commenced radiation on cycle one, day 16 of chemotherapy, and completed the last fraction of radiation on cycle three, day two of chemotherapy, with no unplanned treatment breaks. Cycle four of chemotherapy was completed in July 2020. Toxicities were graded according to CTCAE v5. Acute radiation toxicities included grade 2 urinary urges requiring oxybutynin patches and grade 1 urinary retention. Acute chemotherapy toxicities included grade 1 nausea, constipation, lethargy, and cytopenia (neutropenia, lymphopenia, and anemia). Late toxicities were limited to grade 2 urinary urge and grade 1 urinary retention, which were unchanged from baseline. The patient was followed up in a rotational arrangement between the radiation oncologist, medical oncologist, and urologist, with a three-monthly clinical examination, a three-monthly PSA, a six-monthly CT of the chest, abdomen, and pelvis, and a six-monthly cystoscopy. As of the last follow-up in December 2023, the patient remained in complete remission 41 months after the completion of treatment. The latest PSA was <0.008 ug/L in October 2023.

## Discussion

We present the first case of synchronous primary bladder and primary prostate cancer, treated curatively with definitive chemoradiation therapy, resulting in long-term remission, minimal toxicity, excellent functional outcome, and organ preservation. Concomitant prostatic adenocarcinoma is incidentally found in 24-51% of radical cystoprostatectomy specimens in the course of managing primary bladder cancer [3]. MEDLINE-indexed reports of synchronous localized bladder and prostate cancers have been exclusively managed with radical surgery, with or without perioperative chemotherapy. We were unable to find a case of definitive chemoradiation therapy for synchronous primary bladder and primary prostate cancers, regardless of histological type. Definitive chemoradiotherapy for bladder cancers allows organ preservation in 90% of patients and achieves survival outcomes similar to a surgical approach with radical cystectomy and perioperative chemotherapy [4,5]. Similarly, the PROTECT randomized controlled trial demonstrated equivalent survival outcomes between surgery and radiation for localized prostate cancer [6]. This case report demonstrates the feasibility, safety, and efficacy of definitive chemoradiation therapy for synchronous bladder and prostate cancers, resulting in organ preservation, which presents an appealing alternative to radical cystoprostatectomy for both patients and clinicians.

In addition, this is only the third reported case of synchronous SCCB and prostate adenocarcinoma and the first report describing curative-intent definitive chemoradiation therapy. Liu et al. reported two cases of synchronous SCCB and prostate adenocarcinoma managed curatively with upfront radical cystoprostatectomy and adjuvant chemotherapy (carboplatin/etoposide), with one patient dying at nine months and one alive at 19 months follow-up [7]. Non-urothelial or non-transitional cell histology is found in 53%-55% of bladder cancers, with squamous cells (45%) and micropapillary (26%) comprising the majority of variant histology [8,9]. SCCB is an aggressive and rare diagnosis, with an annual incidence of 0.03 to 0.14 per 100,000 persons, accounting for 0.7%-1% of all bladder cancer diagnoses and conferring the worst prognosis of all bladder cancer histology, with 37% of cases presenting with nodal or distant metastases at diagnosis, compared to 13.6% for urothelial carcinoma [1,2]. While 93% of all small cell carcinomas are pulmonary in origin, the most common extrapulmonary sites include the urinary bladder, prostate, and uterine cervix [10]. However, extrapulmonary small cell carcinoma appears to behave less aggressively than pulmonary small cell carcinoma, as significantly fewer SCCB are diagnosed with de novo extensive stage disease compared to pulmonary small cell carcinoma, and SCCB also exhibits an improved stage-specific prognosis [10,11]. Microscopic appearances of SCCB typically resemble those of other neuroendocrine carcinomas, featuring sheets of high-grade cells with nuclear molding, overlapping nuclei, and dispersed “salt and pepper” chromatin, with about 50% of cases being pure small cell histology and 50% mixed with urothelial carcinoma [12]. Our case showed mixed histology but predominantly small cells (>80%). Pure SCCB has been noted to have a worse prognosis than tumors with a mixed histology [13]. Genomic analysis of bladder cancers identified a propensity for TP53 and RB1 mutations in SCCB versus other histologies, resulting in a worse prognosis [11].

There is limited evidence to guide optimal management of localized SCCB; however, multimodality treatment with platinum-based systemic therapy plus radical cystoprostatectomy or radiation therapy has typically been employed, resulting in a five-year overall survival of 28%-37% [14,15]. There are no randomized controlled trials to guide optimal management. Multimodal treatment appears to provide better survival outcomes than in non-randomized series [14,16]. In one series, radical cystectomy alone resulted in a median overall survival of 1.2 years versus 14.5 years in patients who received neoadjuvant chemotherapy plus radical cystectomy [16]. A National Cancer Database study reported worse survival outcomes with chemotherapy alone compared to chemoradiation or surgery with chemotherapy [14]. A large multicentre British study reported similar survival outcomes between surgery and radiation [15]. Prophylactic cranial irradiation is controversial [9]. A small prospective phase 2 trial reported a 50% risk of developing brain metastases in patients with bulky primary or metastatic disease at diagnosis [17], but in two larger series, only 1.5%-3% of all patients with localized disease at diagnosis experienced intracranial progression [14,16]. 

Survival remains poor in SCCB. Future randomized controlled trials for curative management of localized extrapulmonary small cell carcinoma, including SCCB, are warranted. Questions include identifying the optimal modality for locoregional treatment (surgery or concurrent chemoradiation), the role of prophylactic cranial irradiation, and the role of adjuvant immune checkpoint inhibitors or DOTATE-radioligand therapy. Due to the rarity of extrapulmonary small cell carcinoma, future studies would ideally be multi-institutional and international in design.

## Conclusions

In conclusion, we report the first case of synchronous mixed small cell neuroendocrine carcinoma and high-grade papillary urothelial carcinoma of the urinary bladder and ISUP Grade Group 5 adenocarcinoma of the prostate, managed with definitive concurrent chemoradiation therapy and ADT. This resulted in long-term survival, minimal toxicities, and excellent functional outcomes. Definitive chemoradiation therapy offers a safe and effective, curative-intent organ preservation treatment for localized synchronous prostate and bladder cancers.

## References

[1] Ploeg M, Aben KK, Hulsbergen-van de Kaa CA, Schoenberg MP, Witjes JA, Kiemeney LA (2010). Clinical epidemiology of nonurothelial bladder cancer: analysis of the Netherlands Cancer Registry. J Urol.

[2] Koay EJ, Teh BS, Paulino AC, Butler EB (2011). A surveillance, epidemiology, and end results analysis of small cell carcinoma of the bladder: epidemiology, prognostic variables, and treatment trends. Cancer.

[3] Lopez-Beltran A, Cheng L, Montorsi F, Scarpelli M, Raspollini MR, Montironi R (2017). Concomitant bladder cancer and prostate cancer: challenges and controversies. Nat Rev Urol.

[4] Zlotta AR, Ballas LK, Niemierko A (2023). Radical cystectomy versus trimodality therapy for muscle-invasive bladder cancer: a multi-institutional propensity score matched and weighted analysis. Lancet Oncol.

[5] Softness K, Kaul S, Fleishman A (2022). Radical cystectomy versus trimodality therapy for muscle-invasive urothelial carcinoma of the bladder. Urol Oncol.

[6] Hamdy FC, Donovan JL, Lane JA (2023). Fifteen-year outcomes after monitoring, surgery, or radiotherapy for prostate cancer. N Engl J Med.

[7] Liu Y, Xu H, Wu B, Liu S, Luo Q (2020). Small cell carcinoma of the bladder with coexisting prostate adenocarcinoma: two cases report and literature review. BMC Urol.

[8] Abd El-Latif A, Watts KE, Elson P, Fergany A, Hansel DE (2013). The sensitivity of initial transurethral resection or biopsy of bladder tumor(s) for detecting bladder cancer variants on radical cystectomy. J Urol.

[9] Brocklehurst A, Varughese M, Birtle A (2023). Bladder preservation for muscle-invasive bladder cancer with variant histology. Semin Radiat Oncol.

[10] Dores GM, Qubaiah O, Mody A, Ghabach B, Devesa SS (2015). A population-based study of incidence and patient survival of small cell carcinoma in the United States, 1992-2010. BMC Cancer.

[11] Robertson AG, Kim J, Al-Ahmadie H (2017). Comprehensive molecular characterization of muscle-invasive bladder cancer. Cell.

[12] Park S, Reuter VE, Hansel DE (2019). Non-urothelial carcinomas of the bladder. Histopathology.

[13] Pasquier D, Barney B, Sundar S (2015). Small cell carcinoma of the urinary bladder: a retrospective, multicenter rare cancer network study of 107 patients. Int J Radiat Oncol Biol Phys.

[14] Fischer-Valuck BW, Rao YJ, Henke LE (2018). Treatment patterns and survival outcomes for patients with small cell carcinoma of the bladder. Eur Urol Focus.

[15] Chau C, Rimmer Frcr Y, Choudhury PhD A (2021). Treatment outcomes for small cell carcinoma of the bladder: results from a UK patient retrospective cohort study. Int J Radiat Oncol Biol Phys.

[16] Teo MY, Guercio BJ, Arora A (2022). Long-term outcomes of local and metastatic small cell carcinoma of the urinary bladder and genomic analysis of patients treated with neoadjuvant chemotherapy. Clin Genitourin Cancer.

[17] Siefker-Radtke AO, Kamat AM, Grossman HB (2009). Phase II clinical trial of neoadjuvant alternating doublet chemotherapy with ifosfamide/doxorubicin and etoposide/cisplatin in small-cell urothelial cancer. J Clin Oncol.

